# Supplementary material for: Rehydration rescues Il22−/− mice from lethal Citrobacter rodentium infection
Source: Nat Commun. 2025 Dec 8;17:306. doi: 10.1038/s41467-025-67006-x (PMC12789483; doi:10.1038/s41467-025-67006-x)
Supplement: Supplementary file 1 — Reporting summary [file 41467_2025_67006_MOESM1_ESM.pdf]

nature portfolio

Corresponding author(s): Gad Frankel, Vishwas Mishra

Last updated by author(s): Oct 27, 2025

Reporting Summary

Nature Portfolio wishes to improve the reproducibility of the work that we publish. This form provides structure for consistency and transparency in reporting. For further information on Nature Portfolio policies, see our Editorial Policies and the Editorial Policy Checklist.

Statistics

For all statistical analyses, confirm that the following items are present in the figure legend, table legend, main text, or Methods section.

n/a	Confirmed	
☐	☒	The exact sample size (*n*) for each experimental group/condition, given as a discrete number and unit of measurement
☐	☒	A statement on whether measurements were taken from distinct samples or whether the same sample was measured repeatedly
☐	☒	The statistical test(s) used AND whether they are one- or two-sided<br>*Only common tests should be described solely by name; describe more complex techniques in the Methods section.*
☒	☐	A description of all covariates tested
☐	☒	A description of any assumptions or corrections, such as tests of normality and adjustment for multiple comparisons
☐	☒	A full description of the statistical parameters including central tendency (e.g. means) or other basic estimates (e.g. regression coefficient) AND variation (e.g. standard deviation) or associated estimates of uncertainty (e.g. confidence intervals)
☐	☒	For null hypothesis testing, the test statistic (e.g. *F*, *t*, *r*) with confidence intervals, effect sizes, degrees of freedom and *P* value noted<br>*Give P values as exact values whenever suitable.*
☒	☐	For Bayesian analysis, information on the choice of priors and Markov chain Monte Carlo settings
☒	☐	For hierarchical and complex designs, identification of the appropriate level for tests and full reporting of outcomes
☒	☐	Estimates of effect sizes (e.g. Cohen's *d*, Pearson's *r*), indicating how they were calculated

Our web collection on statistics for biologists contains articles on many of the points above.

Software and code

Policy information about availability of computer code

Data collection	CellQuest Pro (Becton Dickinson), Zen 2.3 (Blue version; Carl Zeiss MicroImaging GmbH, Germany), 12.2 MP, f/1.7, 27mm (wide) Digital Camera (Google PIxel 6a), Omega V5.5 (BMG), Design and Analysis 2.7.0 (Applied Biosystems), SpectroFlo (Cytek Bio), Xcalibur (Thermo Scientific), Real-Time Search–SPS-MS3 (Thermo Scientific)
Data analysis	LEGENDplex Data Analysis Software Suite (Biolegend) , GraphPad Prism 10.4.0 (GraphPad), Zen 2.3 (Blue version; Carl Zeiss MicroImaging GmbH, Germany), Microsoft Office 2024 (Microsoft), MARS data analysis software (BMG), Perseus 2.0.11 (Perseus), FlowJo v10.8.1 software (TreeStar), Phantasus, R studio, R library: tidyverse, janitor, openxlsx, ggrepel, patchwork, Proteome Discoverer 3.0, SequestHT / Comet, Percolator, Perseus 2.0.11, Python (Matplotlib) 3.0.

For manuscripts utilizing custom algorithms or software that are central to the research but not yet described in published literature, software must be made available to editors and reviewers. We strongly encourage code deposition in a community repository (e.g. GitHub). See the Nature Portfolio guidelines for submitting code & software for further information.

Data

Policy information about availability of data

All manuscripts must include a data availability statement. This statement should provide the following information, where applicable:

- Accession codes, unique identifiers, or web links for publicly available datasets
- A description of any restrictions on data availability
- For clinical datasets or third party data, please ensure that the statement adheres to our policy

All data sets generated and/or analyzed during the current study are included in this published article. The Source data file is uploaded with the manuscript. The Source file with all raw values for all the graphs and plots, the raw abundance values for the proteomics analysis and a table with all the materials used is available at FIgshare: https://doi.org/10.6084/m9.figshare.29980600.v2

The mass spectrometry proteomics data have been deposited to the ProteomeXchange Consortium via the PRIDE partner repository with the dataset identifier PXD061225. And can be accessed via the following link: https://www.ebi.ac.uk/pride/archive/projects/PXD061225

Research involving human participants, their data, or biological material

Policy information about studies with human participants or human data. See also policy information about sex, gender (identity/presentation), and sexual orientation and race, ethnicity and racism.

Reporting on sex and gender	*Use the terms sex (biological attribute) and gender (shaped by social and cultural circumstances) carefully in order to avoid confusing both terms. Indicate if findings apply to only one sex or gender; describe whether sex and gender were considered in study design; whether sex and/or gender was determined based on self-reporting or assigned and methods used. Provide in the source data disaggregated sex and gender data, where this information has been collected, and if consent has been obtained for sharing of individual-level data; provide overall numbers in this Reporting Summary. Please state if this information has not been collected. Report sex- and gender-based analyses where performed, justify reasons for lack of sex- and gender-based analysis.*
Reporting on race, ethnicity, or other socially relevant groupings	*Please specify the socially constructed or socially relevant categorization variable(s) used in your manuscript and explain why they were used. Please note that such variables should not be used as proxies for other socially constructed/relevant variables (for example, race or ethnicity should not be used as a proxy for socioeconomic status). Provide clear definitions of the relevant terms used, how they were provided (by the participants/respondents, the researchers, or third parties), and the method(s) used to classify people into the different categories (e.g. self-report, census or administrative data, social media data, etc.) Please provide details about how you controlled for confounding variables in your analyses.*
Population characteristics	*Describe the covariate-relevant population characteristics of the human research participants (e.g. age, genotypic information, past and current diagnosis and treatment categories). If you filled out the behavioural & social sciences study design questions and have nothing to add here, write "See above."*
Recruitment	*Describe how participants were recruited. Outline any potential self-selection bias or other biases that may be present and how these are likely to impact results.*
Ethics oversight	*Identify the organization(s) that approved the study protocol.*

Note that full information on the approval of the study protocol must also be provided in the manuscript.

Field-specific reporting

Please select the one below that is the best fit for your research. If you are not sure, read the appropriate sections before making your selection.

☒ Life sciences ☐ Behavioural & social sciences ☐ Ecological, evolutionary & environmental sciences

For a reference copy of the document with all sections, see nature.com/documents/nr-reporting-summary-flat.pdf

Life sciences study design

All studies must disclose on these points even when the disclosure is negative.

Sample size	No statistical methods were used to pre-determine sample sizes.
Data exclusions	No data were excluded from the analyses.
Replication	All the experiments were reliably reproduced as validated by at least two independent experiments. Some experiments were performed by two different investigators independently with similar results. All attempts of replication were successful. Each dot in graphs represents a mouse.
Randomization	Experimental groups were randomized. Mice were grouped according to genotype and all experiments were performed with age- and

Randomization	sex-matched mice.
Blinding	Investigators were not blinded because the investigator analysing data was also collecting it.

Reporting for specific materials, systems and methods

We require information from authors about some types of materials, experimental systems and methods used in many studies. Here, indicate whether each material, system or method listed is relevant to your study. If you are not sure if a list item applies to your research, read the appropriate section before selecting a response.

Materials & experimental systems

n/a	Involved in the study	
☐	☒	Antibodies
☒	☐	Eukaryotic cell lines
☒	☐	Palaeontology and archaeology
☐	☒	Animals and other organisms
☒	☐	Clinical data
☒	☐	Dual use research of concern
☒	☐	Plants

Methods

n/a	Involved in the study	
☒	☐	ChIP-seq
☐	☒	Flow cytometry
☒	☐	MRI-based neuroimaging

Antibodies

Antibodies used	Purified anti-mouse Ly-6G_Monoclonal antibody (1A8) (BioLegend, 127602), Anti-PCNA antibody [PC10] (Abcam, Ab29), Rabbit polyclonal anti-C. rodentium O152, Anti-E Cadherin antibody [M168] - C-terminal (Abcam, Ab76055), Goat anti-Rat IgG (H+L) Cross-Adsorbed Secondary Antibody, Alexa Fluor™ 647 (Invitrogen, Thermo Fisher Scientific, A-21247), Alexa Fluor® 488 AffiniPure™ Donkey Anti-Rabbit IgG (H+L) (Jackson ImmunoResearch, 711-545-152), Alexa Fluor® 488 AffiniPure® Donkey Anti-Mouse IgG (H+L) (Jackson ImmunoResearch, 715-545-150).
Validation	All antibodies are commercially available and are validated on the manufacturer's website. All antibodies were initially tested and titrated before routine use in the lab.

Animals and other research organisms

Policy information about studies involving animals; ARRIVE guidelines recommended for reporting animal research, and Sex and Gender in Research

Laboratory animals	Mice were housed and bred in dedicated animal facilities of Imperial College London (12h light/dark cycle; 22+/-2°C; 30 to 40% humidity) . All mice were used on a C57BL/6J background. Eight to twelve weeks-old male animals were used for all the experiments.
Wild animals	No wild animals were used
Reporting on sex	Data reported is for male mice only (Fig 2-8). Sex was considered in pilot study design and male and female mice displayed differences in disease progression and therefore, only one sex was used for the study. However, in pilot study, female mice showed similar phenotypes and similar protein abundance changes as analysed using proteomics analysis to male mice and therefore, the findings should be extended to female mice as well.
Field-collected samples	The study did not involve samples collected from the field.
Ethics oversight	All animal work was conducted at Imperial College London (Association for Assessment and Accreditation of Laboratory Animal Care accredited unit) under the auspices of the Animals (Scientific Procedures) Act (UK) 1986 (PP7392693). The animal experiments were approved locally and were designed in agreement with the ARRIVE guidelines and Imperial College London's animal welfare policies, which are founded on the principles of Replacement, Reduction, and Refinement (3Rs)

Note that full information on the approval of the study protocol must also be provided in the manuscript.

Plants

Seed stocks	*Report on the source of all seed stocks or other plant material used. If applicable, state the seed stock centre and catalogue number. If plant specimens were collected from the field, describe the collection location, date and sampling procedures.*
Novel plant genotypes	*Describe the methods by which all novel plant genotypes were produced. This includes those generated by transgenic approaches, gene editing, chemical/radiation-based mutagenesis and hybridization. For transgenic lines, describe the transformation method, the number of independent lines analyzed and the generation upon which experiments were performed. For gene-edited lines, describe the editor used, the endogenous sequence targeted for editing, the targeting guide RNA sequence (if applicable) and how the editor was applied.*
Authentication	*Describe any authentication procedures for each seed stock used or novel genotype generated. Describe any experiments used to assess the effect of a mutation and, where applicable, how potential secondary effects (e.g. second site T-DNA insertions, mosiacism, off-target gene editing) were examined.*

Flow Cytometry

Plots

Confirm that:

☒ The axis labels state the marker and fluorochrome used (e.g. CD4-FITC).

☒ The axis scales are clearly visible. Include numbers along axes only for bottom left plot of group (a 'group' is an analysis of identical markers).

☒ All plots are contour plots with outliers or pseudocolor plots.

☒ A numerical value for number of cells or percentage (with statistics) is provided.

Methodology

Sample preparation	3-cm segments of distal colon from ethically euthanised C57BL/6 mice were excised, washed, and cut opened longitudinally, incubated at 37°C for 20 minutes in a shaking incubator in calcium- and magnesium-free 1X HBSS containing 2% FBS, 10mM EDTA, and 1mM DTT. Following incubation, the cell suspension was centrifuged to separate IECs. Supernatants containing IECs were discarded, and the residual tissue was subjected to enzymatic digestion in RPMI 1640 medium containing 62.5µg/mL Liberase, 50µg/mL DNase I (Sigma-Aldrich), and 2% FBS at 37°C for 40–50 minutes, followed by filtration through 100 µm cell strainer to obtain a single-cell suspension for flow cytometry analysis.
Instrument	Aurora flow cytometer (Cytek Biosciences)
Software	Collection : SpectroFlo software (Cytek Biosciences). Analysis: FlowJo v10.8.1 software (TreeStar)
Cell population abundance	Of the CD45+ cells, Total T cells comprised ~10–15% , CD4⁺ T cells ~8–12%, Th17 cells ~5–8%, Th1 cells ~3–6%, macrophages ~1–2%, Ly6C⁺ cells ~40–50%, Treg cells ~0.5–1%, Th2 cells ~0.5–1%, and neutrophils ~15–25%.
Gating strategy	Described in Figure S5. All antibodies and reagent used for Flow cytometry are available in the Reagent and Resources table available at Figshare: 10.6084/m9.figshare.29980600. Private Link: https://figshare.com/s/ec3ea0f050eedde60d8f

☒ Tick this box to confirm that a figure exemplifying the gating strategy is provided in the Supplementary Information.
