# Supplementary material for: Rehydration rescues Il22−/− mice from lethal Citrobacter rodentium infection
Source: Nat Commun. 2025 Dec 8;17:306. doi: 10.1038/s41467-025-67006-x (PMC12789483; doi:10.1038/s41467-025-67006-x)
Supplement: Supplementary file 2 — Supplementary Information [file 41467_2025_67006_MOESM2_ESM.pdf]

Supplementary Figure S1

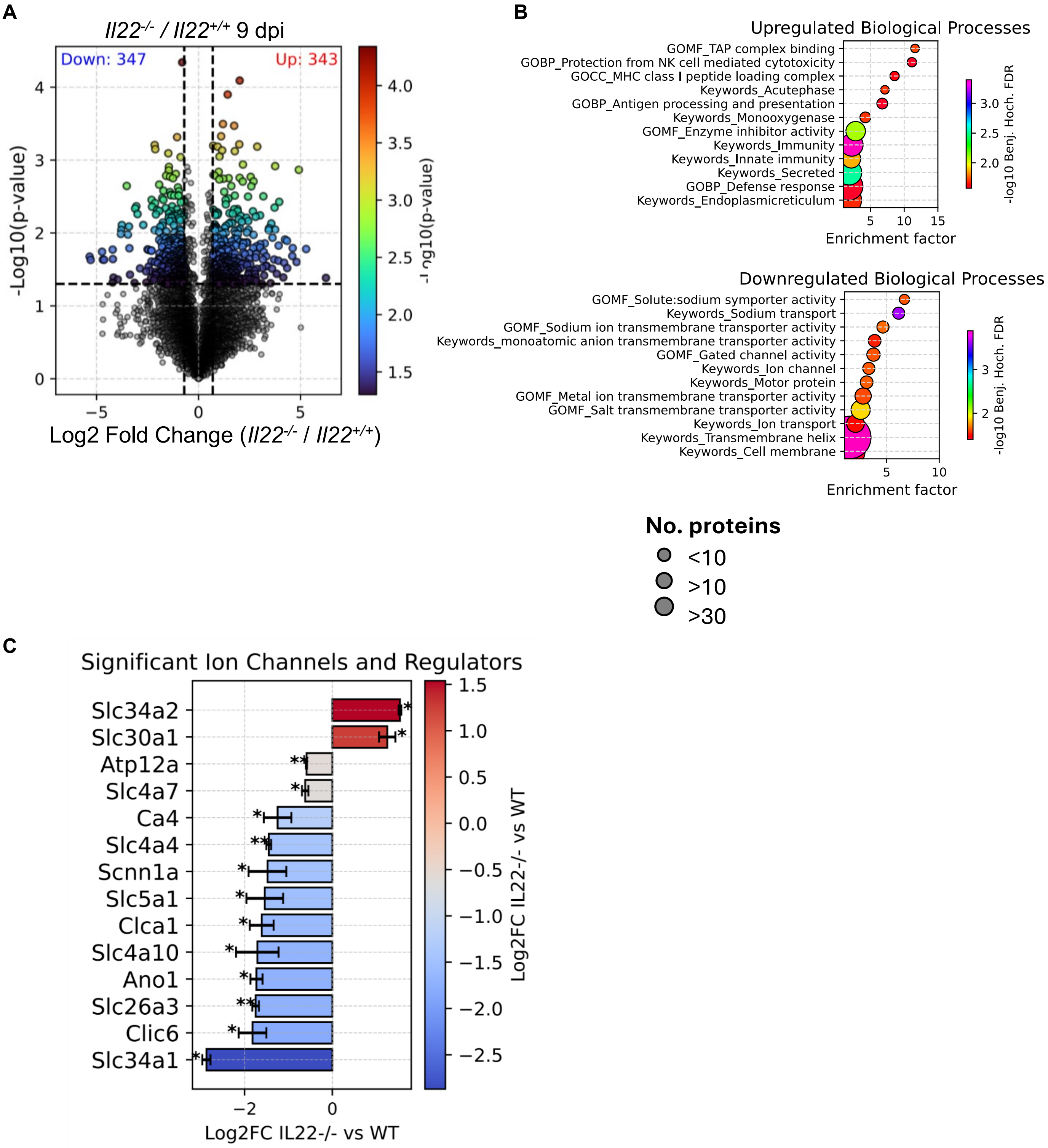

Fig. S1. Proteomic profiling of colonic IECs from *C. rodentium*-infected *Il22*$^{+/+}$ and *Il22*$^{-/-}$ mice. **(A)** Volcano plot showing differentially expressed proteins ($p < 0.05$, $|log2FC| > 0.5$) at 9 dpi. **(B)** Gene set enrichment analysis of differentially regulated pathways, highlighting ion transport–associated pathways. **(C)** Bar graph heat map showing significantly reduced abundance of selected ion transporters in *Il22*$^{-/-}$ mice compared to *Il22*$^{+/+}$ controls. Data represent quantitative proteomics of colonic IECs at 9 dpi from 2 independent experiments with 3-5 mice per experiment.

Supplementary Figure S2

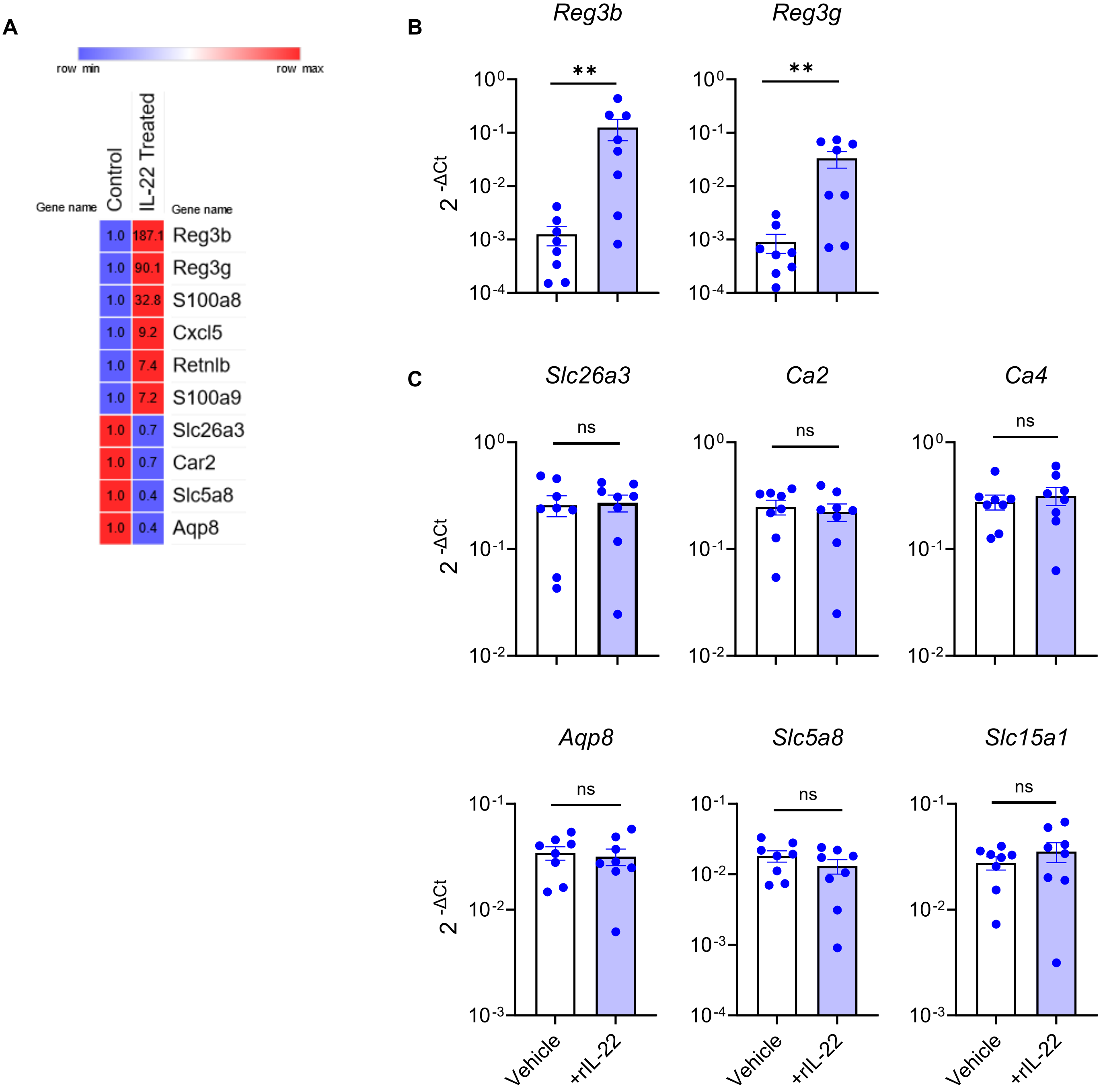

Fig. S2. IL-22 does not directly regulate transcription of ion transporters in the colon. (A) Analysis of publicly available colonic organoid dataset from Powell *et al.,* 2020, treated with rIL-22 showing no induction of ion transporters, IL-22 induced genes were used as control. The numbers show the absolute expression value from the dataset. Also see **Table S2**. **(B-C)** qRT-PCR of *Reg3b*, *Reg3g*, and ion transporter genes in colons of naïve mice treated with rIL-22. Each dot represents an individual mouse. Data represent 2 biological repeats with 4 mice per group per repeat (see Supplementary Data 1). Data shown as Mean ± SEM. P values determined on log2 values using Student's t-test. ns, non-significant; **, $p < 0.01$. Source data with all raw values and exact p values are provided as a Source Data file.

Supplementary Figure S3

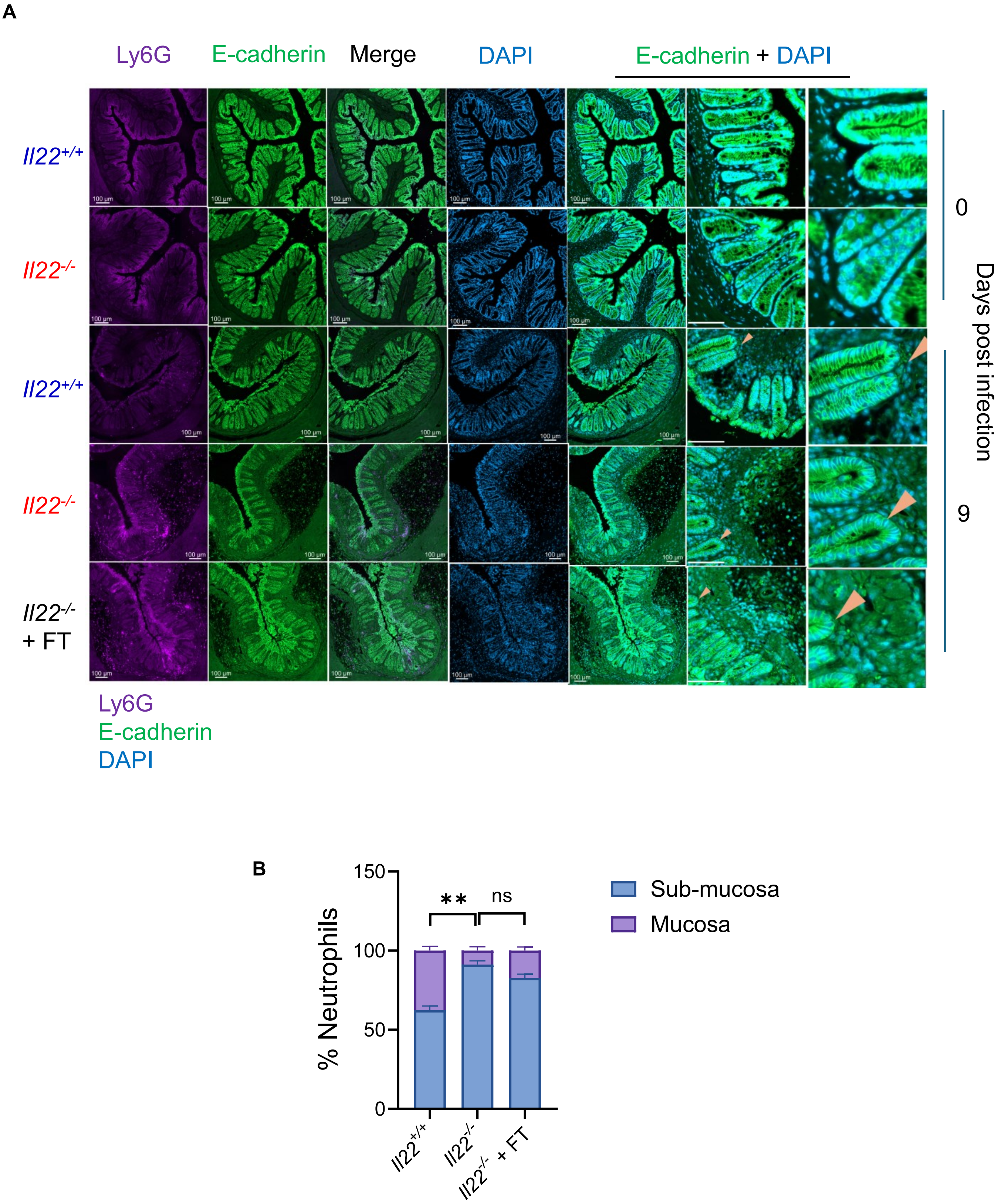

Fig. S3. Neutrophil infiltration and epithelial barrier loss in *C. rodentium*-infected *Il22*$^{-/-}$ mice. (A) Representative immunofluorescence staining of colonic sections from uninfected and *C. rodentium*-infected *Il22*$^{+/+}$ and *Il22*$^{-/-}$ ± FT mice at 9 dpi. Staining includes Ly6G (neutrophils), E-cadherin (epithelial junctions), and DAPI (nuclei). Note, arrows depicting E-cadherin staining the cell boundaries, while DAPI stains the nucleus. Scale bar: 100 µm. Representative of 2 independent experiments, n = 3-4 mice per group. **(B)** Proportion of sub-mucosal and mucosal localisation of the total colonic neutrophils. Data shown as Mean ± SEM. P values determined on the sub-mucosal values using One-way ANOVA with Bonferroni post-test. ns, non-significant; **, $p < 0.01$. Source data with all raw values and exact p values are provided as a Source Data file.

Supplementary Figure S4

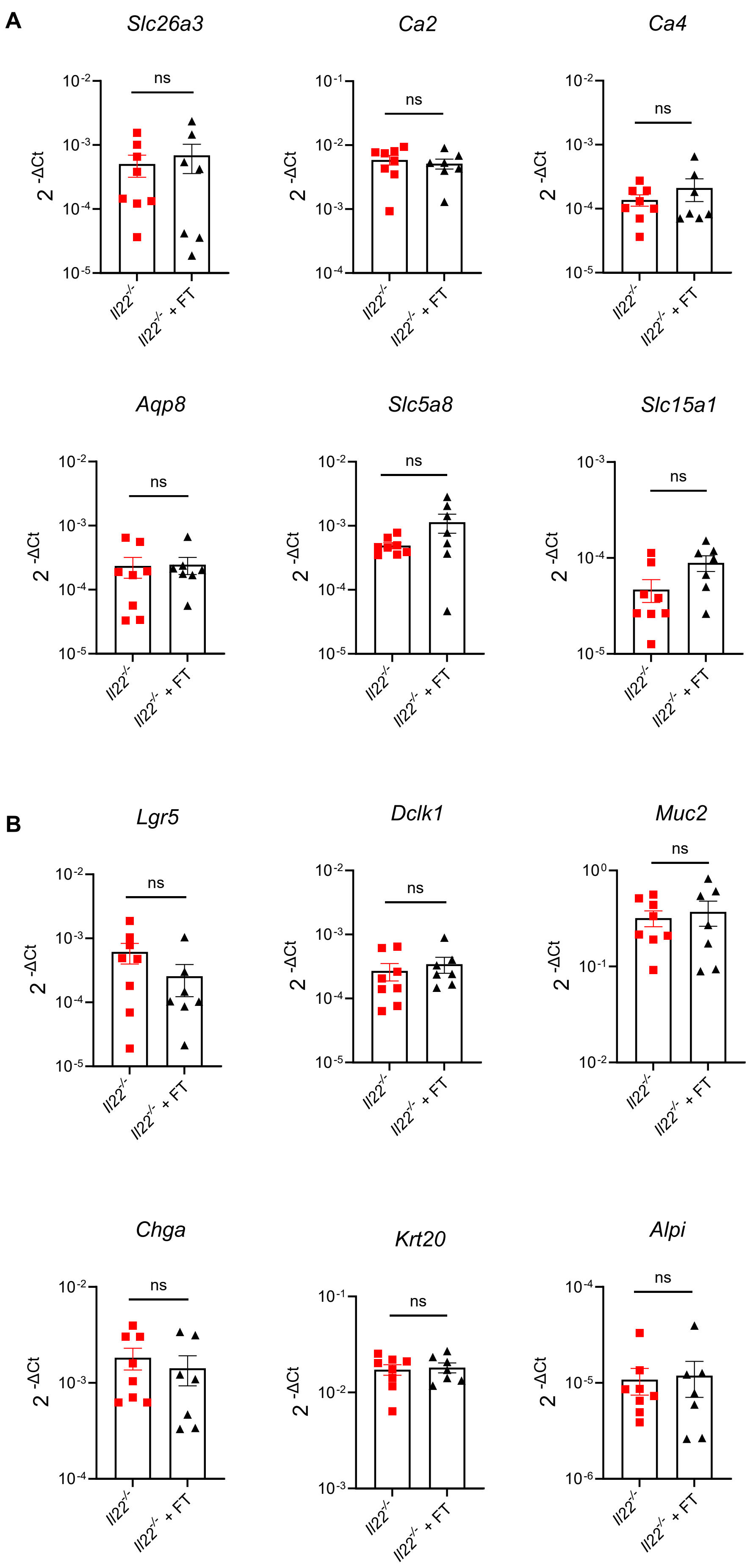

Fig. S4. FT did not alter the expression of colonic ion transporters or epithelial-subset marker genes. (A-B) qRT-PCR analysis of colonic ion transporters **(A)** and epithelial subtype markers: *Lgr5* (stem cells), *Dclk1* (tuft), *Chga* (enteroendocrine), *Muc2* (goblet), *Alpi* and *Krt20* (enterocytes) **(B)** in colonic tissue from *Il22*$^{-/-}$ and *Il22*$^{-/-}$ + FT mice at 9 dpi. Each dot represents one mouse; data for *Il22*$^{-/-}$ is same as used in Fig. 2, refer to 3Rs section in Methods. Data from 2 biological repeats, n = 4 per group. Mean ± SEM shown. P values determined on log2 values using Student's t-test. ns, non-significant. Source data with all raw values and exact p values are provided as a Source Data file.

Supplementary Figure S5

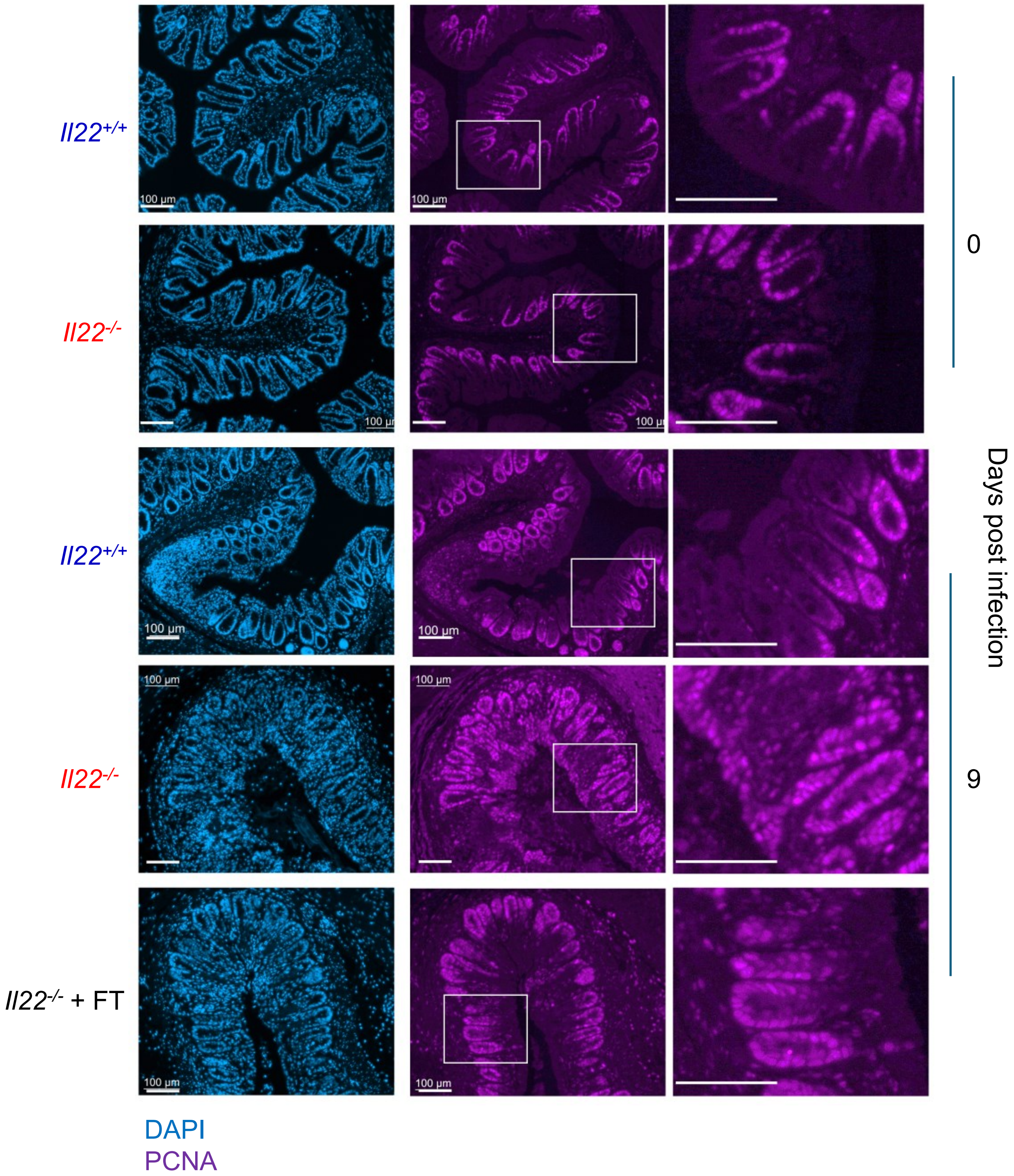

Fig. S5. PCNA staining of colonic sections. Representative PCNA immunofluorescence staining of colonic sections (violet) from uninfected and *C. rodentium*-infected *Il22*$^{+/+}$ and *Il22*$^{-/-}$ ± FT mice at 9 dpi. DAPI used for nuclear staining. Scale bar: 100 µm. Representative of 2 independent experiments, n = 3-4 mice per group.

Supplementary Figure S6

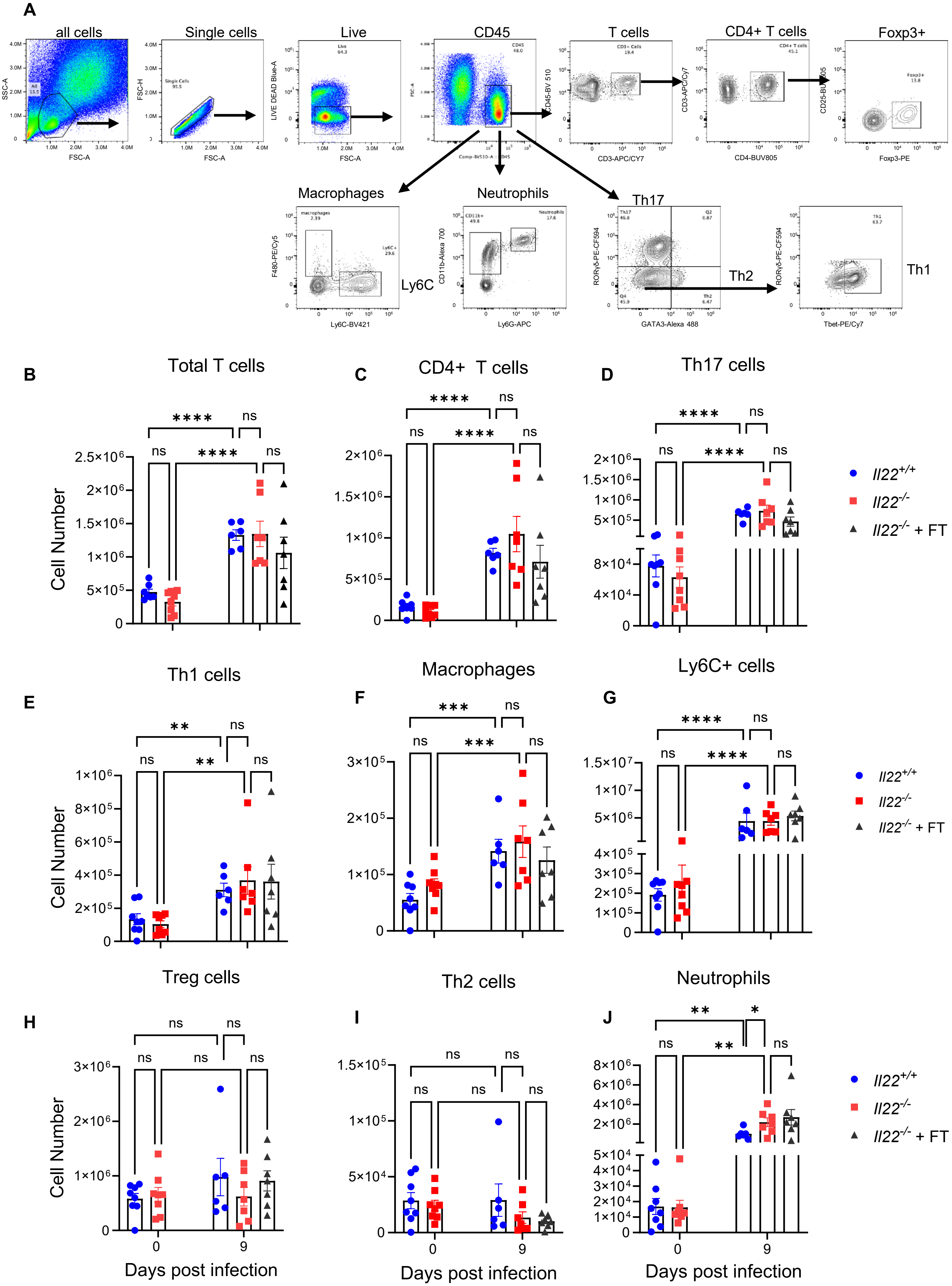

Fig. S6. FT does not alter immune cell composition in the colons of *C. rodentium*-infected mice. **(A)** Gating strategy for colonic immune cell populations by flow cytometry. **(B-J)** Absolute numbers of total T cells **(B)**, CD4+ T cells **(C)**, Th17 cells **(D)**, Th1 cells **(E)**, macrophages **(F)**, Ly6C+ monocytes **(G)**, Treg cells **(H)**, Th2 cells **(I)** and neutrophils **(J)** in $Il22^{+/+}$ and $Il22^{-/-}$ ± FT mice at 0 and 9 dpi. Each dot represents one mouse; data pooled from 2 biological repeats with 3-4 mice per group (See Supplementary Data 1). Mean ± SEM shown. P values determined by Two-way ANOVA with Bonferroni post-test. ns, non-significant; *, $p < 0.05$; **, $p < 0.01$; ***, $p < 0.001$; ****, $p < 0.0001$. Source data with all raw values and exact p values are provided as a Source Data file.

Supplementary Figure S7

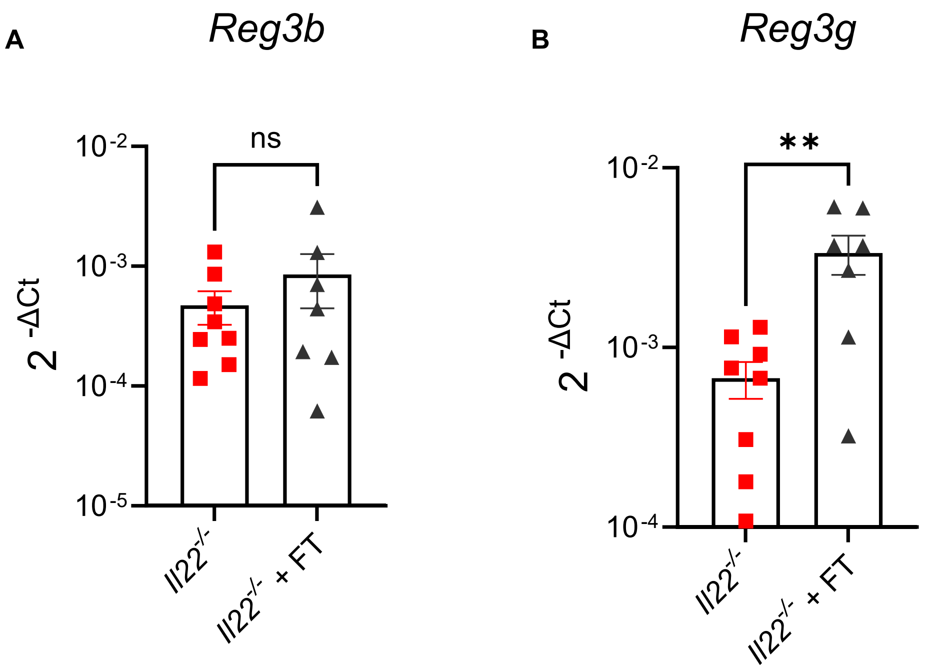

Fig. S7. FT increases Reg3g expression in *Il22*$^{-/-}$ mice. (A-B) qRT-PCR analysis of *Reg3b* **(A)** and *Reg3g* **(B)** in colonic tissue from *Il22*$^{-/-}$ and *Il22*$^{-/-}$ + FT mice at 9 dpi. Each dot represents one mouse; data for *Il22*$^{-/-}$ is same as used in Fig. 2, refer to 3Rs section in Methods. Data from 2 biological repeats, n = 4 per group. Mean ± SEM shown. P values determined on log2 values using Student's t-test. ns, non-significant; **, $p < 0.01$. Source data with all raw values and exact p values are provided as a Source Data file.

Supplementary Figure S8

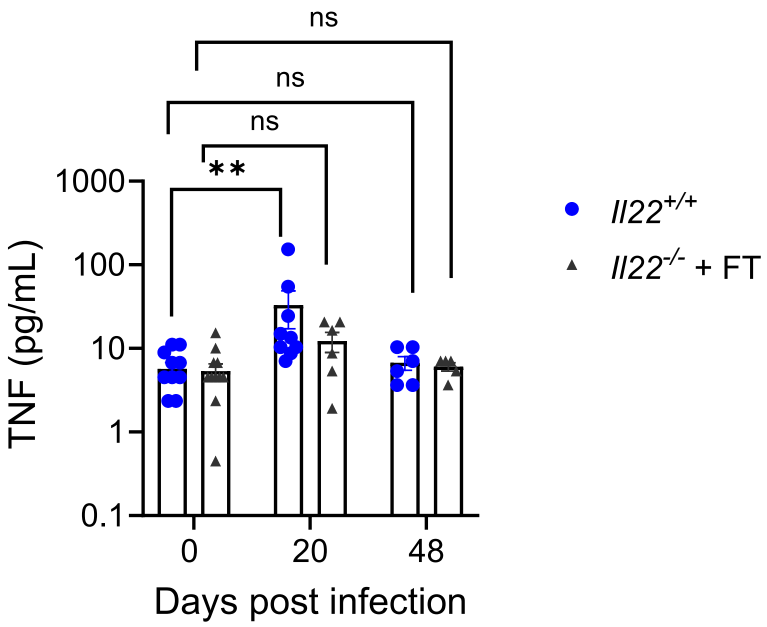

Fig. S8. Cytokine levels during late recovery. TNF levels in *Il22*$^{+/+}$ and *Il22*$^{-/-}$ mice at 20 and 48 dpi compared to uninfected controls. Each dot represents one mouse; data for 0 dpi is same as used in Fig. 6, refer to 3Rs section in Methods. Data pooled from 3 biological repeats with 4-5 mice per group (refer to Supplementary Data 1). Mean ± SEM shown. P values determined by Two-way ANOVA with Bonferroni post-test. ns, non-significant; **, $p < 0.01$. Source data with all raw values and exact p values are provided as a Source Data file.

Supplementary Figure S9

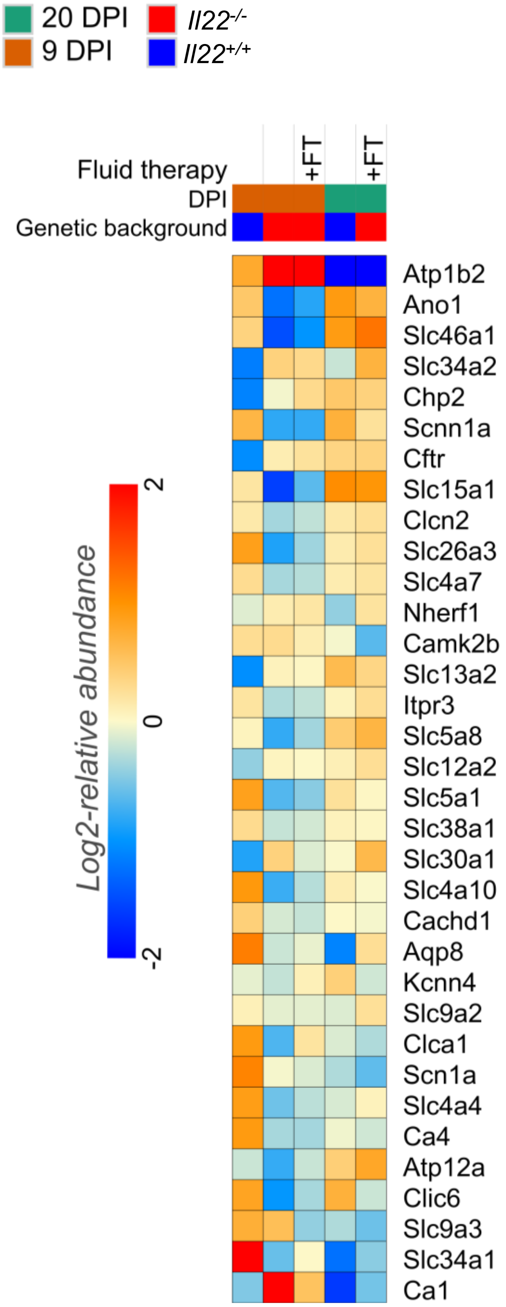

Fig. S9. Recovery of ion transporter expression in *Il22$^{-/-}$* mice at 20 dpi. Heatmap showing restored expression of ion transporters and associated regulators in colonic IECs from *Il22$^{+/+}$* and *Il22$^{-/-}$* mice at 20 dpi. Proteomics data representative of 2 biological repeats with 3-4 mice per group per experiment.

Supplementary Table S1. Compiled data of survival studies of *Il22*$^{-/-}$ mice infected with CR

Mouse strain	Survival range in each figure (days)	% Lethality	Reference
C57BL/6	7 – 13, 6 - 14	90	Zheng *et al., Nat Med,* 2008
C57BL/6	9 – 25, 8 - 25	75	Basu *et al., Immunity,* 2012
C57BL/6	8 – 14, 7 – 13, 7 – 13, 7 – 8, 10 – 13.	100	Xia et al., *Plos Path,* 2019
C57BL/6	7 - 13	100	Zindl *et al., Immunity,* 2022
C57BL/6	9 - 14	100	Liu et al., *Plos Path,* 2024
C57BL/6	12 - 25	91	Melchior *et al., Infect Immun,* 2024
C57BL/6	7 – 14	100	This study

1. Zheng, Y. et al. Interleukin-22 mediates early host defense against attaching and effacing bacterial pathogens. Nat Med 14, 282-289 (2008). https://doi.org:10.1038/nm1720
2. Basu, R. et al. Th22 cells are an important source of IL-22 for host protection against enteropathogenic bacteria. Immunity 37, 1061-1075 (2012). https://doi.org:10.1016/j.immuni.2012.08.024
3. Xia, X. et al. EspF is crucial for Citrobacter rodentium-induced tight junction disruption and lethality in immunocompromised animals. PLoS Pathog 15, e1007898 (2019). https://doi.org:10.1371/journal.ppat.1007898
4. Liu, Y. et al. The gut microbiota-independent virulence of noninvasive bacterial pathogen Citrobacter rodentium. PLoS Pathog 20, e1012758 (2024). https://doi.org:10.1371/journal.ppat.1012758
5. Melchior, K. et al. IL-22-dependent responses and their role during Citrobacter rodentium infection. Infect Immun 92, e0009924 (2024). https://doi.org:10.1128/iai.00099-24

Supplementary Table S2. Fold change in expression of IL-22–inducible genes and ion channels in recombinant IL-22–treated mouse colonic organoids (Powell *et al.,* 2020).

Gene	Fold change	p-value	q-value
Reg3b	187.07	7.21E-15	1.22E-10
Reg3g	90.08	9.77E-13	4.13E-09
S100a8	32.84	3.18E-13	2.69E-09
Cxcl5	9.18	2.00E-12	5.63E-09
Retnlb	7.36	4.76E-10	2.78E-07
S100a9	7.24	3.15E-10	2.22E-07
Slc26a3	0.71	3.45E-04	7.46E-03
Car2	0.71	1.99E-05	9.11E-04
Slc5a8	0.39	5.79E-08	9.98E-06
Aqp8	0.36	9.90E-08	1.42E-05
Alpi	0.35	8.61E-08	1.30E-05

Powell, N. et al. Interleukin-22 orchestrates a pathological endoplasmic reticulum stress response transcriptional programme in colonic epithelial cells. Gut 69, 578-590 (2020). https://doi.org/10.1136/gutjnl-2019-318483

Supplementary Table S3. Estimation of dehydration status and corresponding FT regime for a CR-infected *Il22*$^{-/-}$ mouse (weight ~30grams) during infection

Day post Infection	% body weight loss	Wet food intake	Ruffled coat	Motility score	Posture score	Estimated dehydration %	Estimated volume (µl) of injections/24 h
5	2	++	0	0	0	2	600
6	2.5	++	0	0	0	2.5	750
7	5	+	0	0	0	6	1800
8	5	+	0	0	0	6	1800
9	7	+	0	0	0	8	2400
10	10	+	1	1	0	10	3000
11	10	-	1	2	1	10	3000
12	10	-	2	2	1	10	3000
13	10	-	2	2	1	10	3000
14	10	-	1	2	1	10	3000
15	10	+	1	2	1	10	3000
16	7	+	0	1	0	8	2400
17	5	++	0	1	0	6	1800
18	5	++	0	0	0	6	1800
19	3	++	0	0	0	3	900
20	2	++	0	0	0	2	600

Supplementary Table S4. List of qRT-PCR primers used in the study

Gene name	Fwd Primer sequences (5' to 3')	Rvs Primer sequence (5' to 3')
Reg3b	ATGGCTCCTACTGCTATGCC	GTGTCCTCCAGGCCTCTTT
Reg3g	ATGGCTCCTATTGCTATGCC	GATGTCCTGAGGGCCTCTT
Slc26a3	GCCGTGGTTGGGAACATGA	GCAAATCCTTTGAATGCTCCAG
Ca2	TCCCACCACTGGGGATACAG	CTCTTGGACGCAGCTTTATCATA
Ca4	TACGTGGCCCCCTCTACTG	GCTGATTCTCCTTACAGGCTCC
Aqp8	TGTGTAGTATGGACCTACCTGAG	ACCGATAGACATCCGATGAAGAT
Slc5a8	CGGGACATCGGCAGTTTTG	CTGCGACCGCCCATAAGAA
Slc15a1	CCGGCACACCCTTCTAGTG	TGGCGTTGTGACTGGTGAC
Lgr5	ACCCGCCAGTCTCCTACATC	GCATCTAGGCGCAGGGATTG
Dclk1	TCCACCGGAATTGAACTCGG	GGGAGCGAACAGTCTCAGA
Chga	CAGCTCGTCCACTCTTTCCG	CCTCTCGTCTCCTTGGAGGG
Muc2	ATGCCCACCTCCTCAAAGAC	GTAGTTTCCGTTGGAACAGTGAA
Alpi	AGGACATCGCCACTCAACTC	GGTTCCAGACTGGTTACTGTCA
Krt20	CAACGGATCGGACCTGTTTG	AGCGCACTTTTTCTAGGTAGTTT
