# Supplementary material for: Rehydration rescues Il22−/− mice from lethal Citrobacter rodentium infection
Source: Nat Commun. 2025 Dec 8;17:306. doi: 10.1038/s41467-025-67006-x (PMC12789483; doi:10.1038/s41467-025-67006-x)
Supplement: Supplementary file 4 — Description of Additional Supplementary File [file 41467_2025_67006_MOESM4_ESM.pdf]

Description of Additional Supplementary Files

Supplementary Data 1: Details of exact number of mice used for all experiments in Fig 1-8, S2-S4, S6-7
